# ﻿Comprehensive molecular and morphological analysis of *Brachystemmacalycinum* and *Stellariaovatifolia* in the tribe Alsineae (Caryophyllaceae)

**DOI:** 10.3897/phytokeys.220.96126

**Published:** 2023-02-22

**Authors:** Wen-Qiao Wang, Zhi-Wei Su, Zhong-Hui Ma

**Affiliations:** 1 College of Agriculture, State Key Laboratory for Conservation and Utilization of Subtropical Agro-bioresources, National Demonstration Center for Experimental Plant Science Education, Traditional Chinese Herbal Medicine Resources and Agriculturalization Research Institute, Guangxi University, Nanning 530004, China Guangxi University Nanning China; 2 Institute of Marine Drugs, Guangxi University of Chinese Medicine, Nanning 530200, China Guangxi University of Chinese Medicine Nanning China

**Keywords:** Alsineae, *Brachystemma*, molecular phylogeny, *Stellaria*

## Abstract

Over the course of the recent decade, the composition of Alsineae has been drastically changed by means of molecular phylogeny. However, the genus *Brachystemma* has not been sampled in any of the previous studies, and its phylogenetic position is still pending. In addition, the related species *Stellariaovatifolia*, which has at times been placed in *Brachystemma*, *Schizotechium*, or *Stellaria*, has also not been sampled. Here, nuclear ribosomal internal transcribed spacer (ITS) and four plastid regions (*trnL-F*, *matK*, *rbcL*, *rps16*) were used to conduct phylogenetic analyses within Caryophyllaceae and the tribe Alsineae. Ancestral characters (petal margin and number of seeds) were reconstructed in the tribe Alsineae based on the phylogenetic results. Our results indicate that *Brachystemma* is nested in the tribe Alsineae and forms a monophylum with *S.ovatifolia*, and apically lobed petals and numerous seeds may be the ancestral characters in the tribe Alsineae. Based on our study, *Stellariaovatifolia* should be considered within *Brachystemma*, and *Brachystemma* is clearly a separate genus and now includes two species.

## ﻿Introduction

The family Caryophyllaceae has traditionally been divided into three subfamilies (Lu et al. 2001). Recently, a new classification system has been proposed based on molecular and morphological evidence in Caryophyllaceae, and eleven tribes were recognized (Harbaugh et al. 2010; Greenberg and Donoghue 2011).

The tribe Alsineae in a traditional sense contained 12 genera including *Arenaria* L., *Brachystemma* D.Don, *Cerastium* L., *Holosteum* L., *Lepyrodiclis* Fenzl, *Minuartia* Loefl., *Moehringia* L., *Myosoton* Moench, *Pseudostellaria* Pax, *Sagina* L., *Stellaria* L., and *Thylacospermum* Fenzl (Lu et al. 2001). However, molecular studies have revealed that the traditional tribe Alsineae is polyphyletic (Harbaugh et al. 2010; Greenberg and Donoghue 2011). To date, the tribe Alsineae now consists of 16 genera including five new genera (*Engellaria* Iamonico, *Hartmaniella* M.L.Zhang & Rabeler, *Nubelaria* M.T.Sharples & E.A.Tripp, *Rabelera* M.T.Sharples & E.A.Tripp, and *Shivparvatia* Pusalkar & D.K.Singh), three reinstated genera (*Adenonema* Bunge, *Odontostemma* Benth. ex G.Don, and *Schizotechium* (Fenzl) Rchb.), and eight originally accepted genera: *Cerastium*, *Dichodon* (Bartl. ex Rchb.) Rchb., *Holosteum*, *Lepyrodiclis*, *Mesostemma* Vved., *Moenchia* Ehrh., *Pseudostellaria*, and *Stellaria* (Keshav and Kumar 2015; Sadeghian et al. 2015; Pusalkar and Srivastava 2016; Zhang et al. 2017; Sharples and Tripp 2019; Iamonico 2021; Yao et al. 2021; Arabi et al. 2022). *Brachystemma* morphologically related to other Alsineae still lacks comprehensive molecular and morphological study.

**Figure 1.**
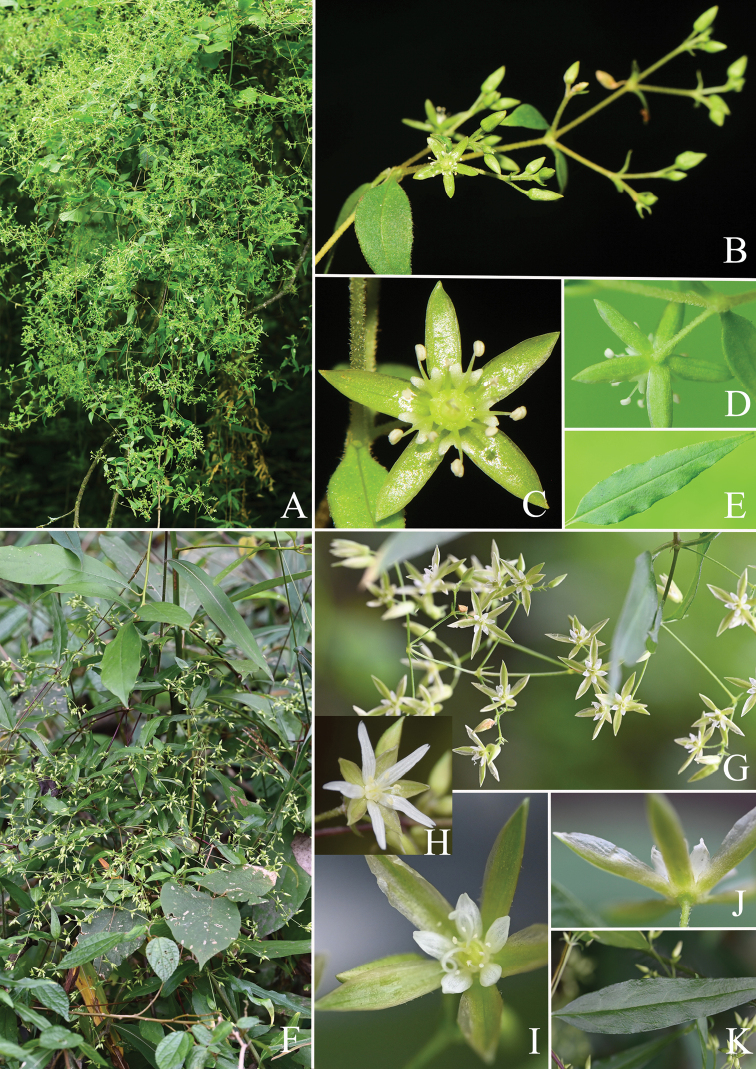
Morphological comparisons between *Stellariaovatifolia* (**A–E**) and *Brachystemmacalycinum* (**F–K**) **A, F** habit **B, G** inflorescence **C, H, I** flower (**H** the flower of *Brachystemmacalycinum* 3) **D, J** sepal **E, K** leaf.

*Brachystemmaovatifolium* Mizushima was first published in 1955 and is related to *Brachystemmacalycinum* D.Don (Mizushima 1955; Fig. 1 in present paper). Subsequently, Mizushima transferred it to *Stellaria* as *Stellariaovatifolia* (Mizushima) Mizushima due to its two-lobed petals and similar seed morphology (Mizushima 1966), which was also accepted by Flora Reipublicae Popularis Sinicae (Wu and Ke 1996) and Flora of China (Shilong and Rabeler 2001). In the first book, it was incorporated into sect. Schizothecium Fenzl of *Stellaria*, together with *S.delavayi* Franch. and *S.monosperma* Buch.-Ham. ex D.Don (Wu and Ke 1996). Recently, Stellariasect.Schizotechium has been raised into a separate genus, *Schizotechium* (Pusalkar and Srivastava 2016), and the new combination *Schizotechiummonospermum* (Buch.-Ham. ex D.Don) Pusalkar & S.K.Srivast. was proposed based on morphological studies (Pusalkar and Srivastava 2016). The molecular studies also indicated that *Stellariamonosperma* was far from the core *Stellaria* and nested within *Schizotechium* (Greenberg and Donoghue 2011; Sharples and Tripp 2019; Arabi et al. 2022). Although *Stellariaovatifolia* was hypothesized to be part of *Schizotechium* (Pusalkar and Srivastava 2016), it has never been sampled and has at times been placed in *Brachystemma*, *Schizotechium*, or *Stellaria*, and its phylogenetic position is still pending.

In this study, we conducted a combined molecular and morphological analysis in order to (1) confirm the phylogenetic position of *Brachystemma*; (2) clarify the relationship of *Stellariaovatifolia* among *Stellaria*, *Schizotechium*, and *Brachystemma*; (3) estimate the character evolution of seed number and petal margin in the tribe Alsineae.

## ﻿Methods

### ﻿Taxon sampling and DNA sequencing

The samples of *Brachystemmacalycinum* and *Stellariaovatifolia* were collected from silica-dried leaves tissue, and the vouchers were deposited in the herbarium of the College of Agriculture, Guangxi University (**GAUA**) and the detailed information is shown in Suppl. material 1. The total DNA of the samples were extracted by the CTAB protocol (Maddison and Maddison 2014). The PCR amplification of ITS [5F (White et al. 1990), 4R (White et al. 1990)], *matK* [390F (Smissen et al. 2002), 1440R (Smissen et al. 2002)], *rbcL* [1F (Kress and Erickson 2007), 724R (Kress and Erickson 2007)], *rps16* [F (Popp and Oxelman 2001), R (Popp and Oxelman 2001)], *trnL-F* [C (Taberlet et al. 1991), F (Taberlet et al. 1991)] were performed as above cited. The sequencing of PCR products was performed by the Beijing Genomics Institute (BGI). Newly generated sequences are available in GenBank (https://www.ncbi.nlm.nih.gov/), and their accession numbers (in bold) and the sequences of Caryophyllaceae members downloaded from GenBank are listed in Table 1. The absent sequences were coded as missing data.

**Table 1.** List of sampled taxa and their GenBank accession numbers of sequences. The arrangement of sequences in the table shows sequences used to generate the trees shown in Fig. 3A, B. Sequences in bold were generated in this study.

Taxon	GenBank accession numbers				
	*nr* ITS	*trnL-F*	*matK*	*rps16*	*rcbL*
**A. Sequences used to generate Caryophyllaceae tree (Fig. 3A)**					
*Agrostemmagithago* L.	JN589107	EU221639	FJ589503	Z83154	KM360618
*Arenarialanuginosa* (Michx.) Rohrb.	MZ388084	FJ404968	MH037652	FJ404891	MH028838
***Brachystemmacalycinum* D.Don**	OP594537	OP595543	OP595548	OP595553	OP595558
*Cerastiumpusillum* Ser.	JN589112	JN589683	JN589226	-	-
*Corrigiolaandina* Planch. & Triana	JN589136	JN589707	JN589253	-	-
*Dianthusarmeria* L.	GU440780	FJ404980	KP210382	FJ404903	MG249427
*Dianthuscaryophyllus* L.	JN589053	MT312520	KU722867	KU904222	M77699
*Eremogonebryophylla* (Fernald) Pusalkar & D.K.Singh	MK341317	MK341206	MK341382	MK341262	-
*Eremogonegypsophiloides* Fenzl	KP148920	-	-	KP149022	-
*Gymnocarposprzewalskii* Bunge ex Maxim.	AJ310971	-	-	MH917997	-
*Gypsophilapaniculata* L.	KX183986	KX183948	KX183906	FJ404908	MG547371
*Holosteummarginatum* C.A.Mey.	JN589093	JN589732	JN589261	-	-
*Lepyrodiclisholosteoides* (C.A.Mey.) Fenzl ex Fisch. & C.A.Mey.	MH808296	FJ404989	FJ404840	KP149043	JQ933385
*Lychniswilfordii* (Regel) Maxim.	KX757649	-	-	LC423834	-
*Moehringialateriflora* (L.) Fenzl	JX274536	FJ405000	MK520325	FJ404924	MN623790
*Moehringiamacrophylla* (Hook.) Fenzl	MF964022	FJ405001	KY952464	FJ404925	MF963280
*Polycarpontetraphyllum* (L.) L.	HE586018	FJ405009	MF963465	FJ404932	HM850271
*Sabulinadouglasii* (Fenzl ex Torr. & A.Gray) Dillenb. & Kadereit	KF737459	FJ404992	FJ404842	FJ460221	-
*Saginajaponica* (Sw.) Ohwi	LC634109	-	MK435791	-	MN204811
*Schiedeaglobosa* H.Mann	AY517663	FJ405014	DQ907804	FJ404938	DQ907750
*Schizotechiumjamesianum* (Torr.) Arabi, Rabeler & Zarre	KX158306	FJ405010	KX158343	KX158417	KX158380
*Sileneaprica* Turcz. ex Fisch. & C.A.Mey.	KX757336	FN821322	MH658952	LC423907	KX158399
*Spergulaarvensis* L.	JX274532	KY616142	JN894908	KY513576	KM360994
***Stellariaovatifolia* (Mizushima) Mizushima**	OP594536	OP595542	OP595547	OP595552	OP595557
*Stellariavestita* Kurz	MH117776	EU785988	MH116882	-	MH116433
**Outgroup**					
*Celosiaargentea* L.	KY968928	LT993045	MH767769	FJ404898	AF206747
**B. Sequences used to generate Alsineae tree (Fig. 3B)**					
***Brachystemmacalycinum* D.Don 1**	OP594537	OP595543	OP595548	OP595553	OP595558
***Brachystemmacalycinum* D.Don 2**	OP594538	OP595544	OP595549	OP595554	OP595559
***Brachystemmacalycinum* D.Don 3**	OP594539	OP595545	OP595550	OP595555	OP595560
*Cerastiumarvense* L.	MH219805	FJ404976	AY936295	MH243535	JX848446
*Cerastiumbrachypetalum* Pers.	-	-	-	-	KF997372
*Cerastiumdavuricum* Fisch. ex Spreng.	KX158321	-	KX158358	KX158432	KX158395
Cerastiumdichotomumsubsp.inflatum (Link) Cullen	KX158322	-	KX158359	KX158433	KX158396
*Cerastiumdinaricum* Beck & Szyszył.	KJ716515	KJ716526	-	-	-
*Cerastiumfontanum* Baumg.	GU444015	FJ404977	KX821263	FJ404899	KF602216
*Cerastiumfurcatum* Cham. & Schltdl.	MH117479	-	MH116578	-	MH116103
*Cerastiumlatifolium* L.	-	AY521301, AY521348	-	-	KF602212
*Cerastiumpusillum* Ser.	JN589112	JN589683	JN589226	-	-
*Cerastiumsubtriflorum* Dalla Torre & Sarnth.	MH537035	KJ716527	-	-	-
*Cerastiumszechuense* F.N.Williams	JN589116	JN589674	-	-	-
*Cerastiumtomentosum* L.	JN589031	AY521310, AY521357	JN589244	MH243538	KF997321
*Dichodoncerastoides* (L.) Rchb.	MH219812	AY521340, AY521388	-	MH243542	MG249356
*Dichodondubium* (Bastard) Ikonn.	MH219815	AY521341, AY521389	-	MH243544	-
*Hartmaniellaoxyphylla* (B.L.Rob.) M.L.Zhang	KX158311	-	KX158348	KX158422	KX158385
*Hartmaniellasierra* (Rabeler & R.L.Hartm.) M.L.Zhang	KX158314	-	KX158351	KX158425	KX158388
*Holosteummarginatum* C.A.Mey.	JN589093	JN589732	JN589261	-	-
*Holosteumumbellatum* L.	JN589051	JN589655	MK520188	FJ404909	MK525977
*Lepyrodiclisholosteoides* (C.A.Mey.) Fenzl ex Fisch. & C.A.Mey.	MH808296	FJ404989	FJ404840	KP149043	JQ933385
*Mesostemmadichotomum* (L.) Arabi, Rabeler & Zarre	MT624581	-	-	MT624662	-
*Mesostemmakotschyanum* (Fenzl ex Boiss.) Vved.	MT624582	-	-	MT624664	-
*Mesostemmaperfoliatum* (Rech.f.) Rech.f.	MT624583	-	-	MT624665	-
*Mesostemmaplatyphyllum* Rech.f.	MT624584	-	-	MT624666	-
*Moenchiaerecta* (L.) G.Gaertn., B. Mey. & Scherb.	JN589103	FJ405002	JN895271	FJ404926	JN892479
*Odontostemmabarbatum* (Franch.) Sadeghian & Zarre	KP148852	-	-	-	-
*Odontostemmatrichophorum* (Franch.) Sadeghian & Zarre	AY936243	-	-	-	-
*Pseudostellariaheterophylla* (Miq.) Pax	KX158334	EU785992	KX158371	KX158445	KX158408
*Pseudostellariajaponica* (Korsh.) Pax	KX158307	-	KX158344	KX158418	KX158381
*Pseudostellariamaximowicziana* (Franch. & Sav.) Pax	KX158309	-	KX158346	KX158420	KX158383
*Pseudostellariatianmushanensis* G.H.Xia & G.Y.Li	KX158318	-	KX158355	KX158429	KX158392
*Pseudostellariatibetica* Ohwi	KX158317	-	KX158354	KX158428	KX158391
*Rabeleraholostea* (L.) M.T.Sharples & E.A.Tripp	KX183997	JN589664	KX183916	MH243549	FJ395575
*Schizotechiumamericanum* (Standl.) Arabi, Rabeler & Zarre	KX158335	JN589675	KX158372	KX158446	KX158409
*Schizotechiumjamesianum* (Torr.) Arabi, Rabeler & Zarre 1	KX158306	FJ405010	KX158343	KX158417	KX158380
*Schizotechiumjamesianum* (Torr.) Arabi, Rabeler & Zarre 2	JN589048	-	-	KX158417	-
*Schizotechiummonospermum* (Buch.-Ham. ex D.Don) Pusalkar & S.K.Srivast 1	MT624596	-	-	MT624676	-
*Schizotechiummonospermum* (Buch.-Ham. ex D.Don) Pusalkar & S.K.Srivast 2	MT624595	-	-	MT624675	-
*Schizotechiumturkestanicum* (Schischk.) Arabi, Rabeler & Zarre	MT624597	-	-	MT624677	-
*Shivparvatiaciliolata* (Edgew. & Hook.f.) Pusalkar & D.K.Singh	KP148859	-	-	-	-
*Shivparvatiaglanduligera* (Edgew.) Pusalkar & D.K.Singh	KP148867	-	-	-	-
*Shivparvatiastracheyi* (Edgew.) Pusalkar & D.K.Singh	KP148898	-	-	-	-
*Stellariaalsine* Grimm	AY438312	EU785987	HM850778	-	HM850385
*Stellariaaquatica* (L.) Scop.	AY594303	FJ405004	JN894058	MH243547	KM360890
*Stellariaborealis* Bigelow	JN589064	JN589713	JN589285	-	MG247728
*Stellariachinensis* Regel	JN589133	EU785990	JN589241	-	-
*Stellariacorei* Shinners	JN589046	JN589715	JN589300	-	-
*Stellariacrassifolia* Ehrh.	JN589071	JN589701	KC475924	-	KC484145
*Stellariacuspidata* Willd. ex D.F.K.Schltdl.	JN589099	JN589641	JN589268	FJ404952	-
*Stellariagraminea* L.	AY594304	JN589687	MK520714	MH243548	KM360998
*Stellarialongifolia* Muhl. ex Willd.	JN589146	GQ245567	MK520715	-	JX848448
*Stellarialongipes* Goldie	JN589086	JN589672	KC475949	-	JX848449
*Stellariamedia* (L.) Vill.	MK044722	EU785989	HM850779	Z83152	AF206823
*Stellarianemorum* L.	AY936246	HM590349	AY936298	-	JN893484
***Stellariaovatifolia* (Mizushima) Mizushima 1**	OP594536	OP595542	OP595547	OP595552	OP595557
***Stellariaovatifolia* (Mizushima) Mizushima 2**	OP594540	OP595546	OP595551	OP595556	OP595561
*Stellariapalustris* Ehrh. ex Hoffm.	JN589080	-	MK520716	KX158438	KX158401
*Stellariapubera* Michx.	JN589127	FJ405027	FJ404878	-	KP643834
*Stellariasoongorica* Roshev.	KX158328	-	MF158660	KX158439	KX158402
*Stellariaumbellata* Turcz.	JN589109	JN589737	JN589254	-	MG246195
*Stellariavestita* Kurz	MH117776	EU785988	MH116882	-	MH116433
**Outgroup**					
*Arenariaserpyllifolia* L.	KX158320	FJ404972	KX158357	KX158431	KX158394
*Arenarialanuginosa* (Michx.) Rohrb.	MZ388084	FJ404968	MH037652	FJ404891	MH028838
*Moehringiamacrophylla* (Hook.) Fenzl	MF964022	FJ405001	KY952464	FJ404925	MF963280
*Sabulinadouglasii* (Fenzl ex Torr. & A.Gray) Dillenb. & Kadereit	KF737459	FJ404992	FJ404842	FJ460221	-
*Saginajaponica* (Sw.) Ohwi	LC634109	-	MK435791	-	MN204811

### ﻿Phylogenetic analyses

**Figure 2.**
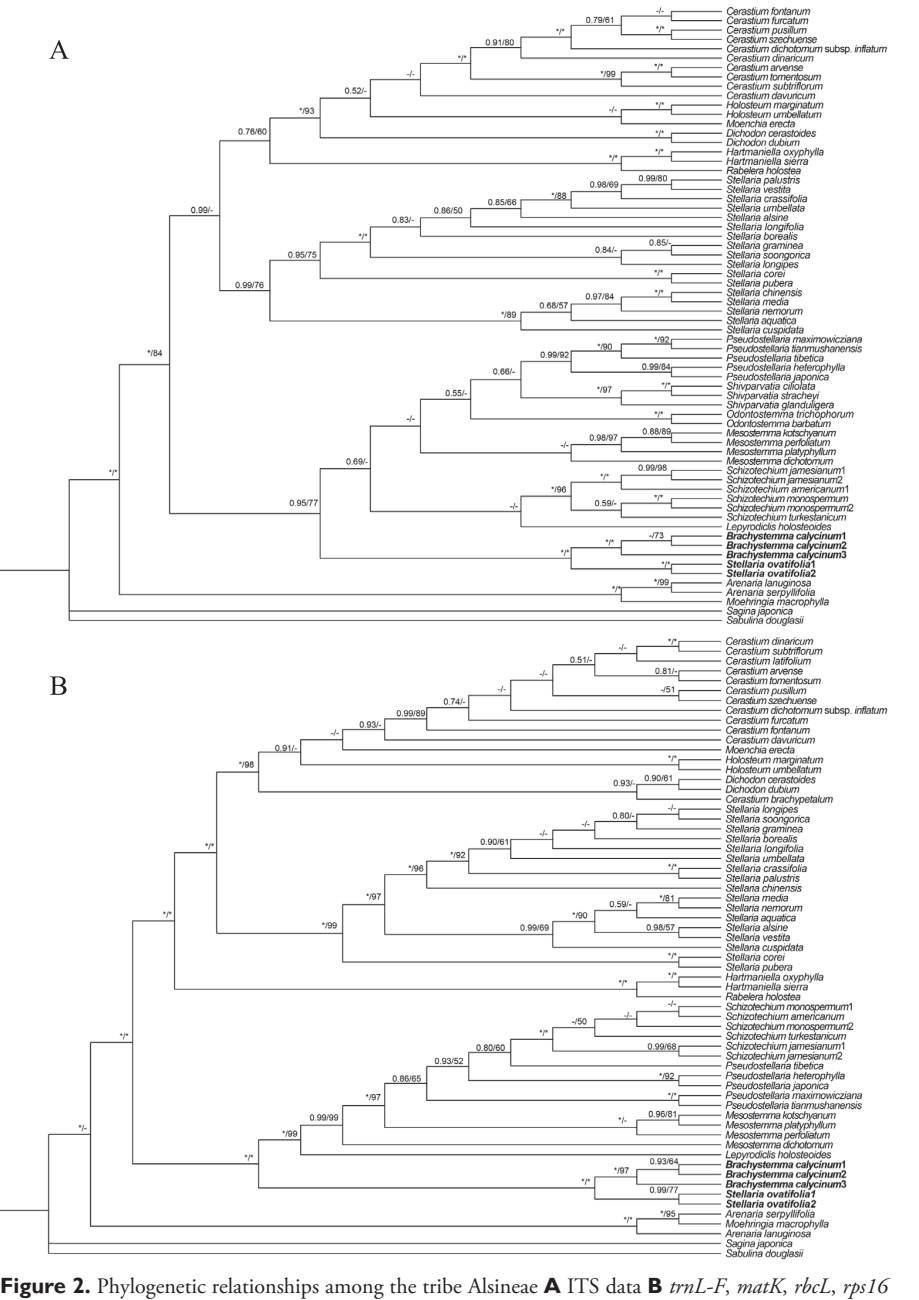
Phylogenetic relationships among the tribe Alsineae**A**ITS data **B***trnL-F*, *matK*, *rbcL*, *rps16* combined data. The numbers on the nodes are Bayesian posterior probabilities (PP > 0.5), maximum likelihood bootstrap percentages (BS > 50%), respectively. “*” indicates that the node is PP = 1.00/BS = 100%, “-” indicates that the node PP < 0.5/ BS < 50%.

Sequences alignment were performed with MAFFT v.7.313 (Katoh and Standley 2013). Phylogenetic analyses were conducted separately on the nuclear ribosomal internal transcribed spacer (ITS) and plastid regions (*matK*, *rbcL*, *trnL-F*, and *rps16*) and then combined; no notable incongruence was found (Fig. 2). The Bayesian Inference (BI) trees were constructed using MRBAYES 3.2.6 (Ronquist and Huelsenbeck 2003), and the maximum likelihood (ML) trees were constructed by RAXML-HPC2 (Stamatakis 2006). ML trees were constructed on CIPRES Science Gateway (Miller et al. 2010) under the GTRGAMMA model with 1,000 bootstrap replicates and default values for the remaining parameters. In Bayesian inference analysis, PARTITIONFINDER v.2.1.1 (Lanfear et al. 2016) was applied to selected models of nucleotide substitution under the Akaike Information Criterion (AIC). Selected models consisted of SYM+I+G for ITS, GTR+G for *matK*, *trnL-F*, and *rps16*, HKY+I+G for *rbcL*. Each Markov chain Monte Carlo (MCMC) analysis was run for 2,000,000 generations with the tree sampled every 100 generations. The first 25% trees of each run as burn-in were discarded.

### ﻿Ancestral characters

Two morphological characters (petal margin and number of seeds) which were diagnostic characters in *Brachystemma* were selected to reconstruct the ancestral characters in the tribe Alsineae. MESQUITE v.3.6 (Maddison and Maddison 2014) was used to reconstruct the ancestral characters with default parameters, using the ML tree from the combined tree. Morphological characters were coded as the following: (a) the petals are entire or emarginate (coded as 0), apex lobed (less than 1/2 the length of the petals) (1), deeply lobed (longer than 1/2 the length of the petals) (2); (b) the number of seeds in a capsule is 1–3 (0), 4–6 (1), more than 6 (2) (Lu et al. 2001; Arabi et al. 2022).

## ﻿Results

### ﻿Phylogenetic analyses

**Figure 3.**
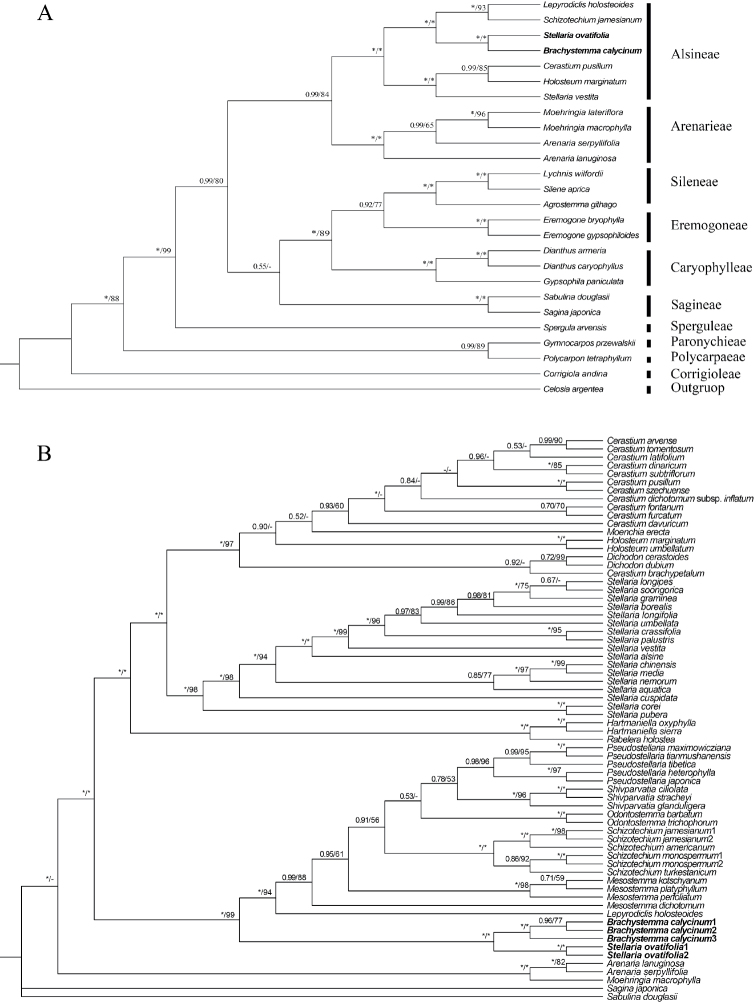
Phylogenetic relationships among the Caryophyllaceae (**A**) and the tribe Alsineae (**B**). Phylogenetic trees were conducted by ITS, *trnL-F*, *matK*, *rbcL*, *rps16* combined sequences. The numbers on the nodes are Bayesian posterior probabilities (PP > 0.5), maximum likelihood bootstrap percentages (BS > 50%), respectively. “*” indicates that the node is PP = 1.00/BS = 100%, “-” indicates that the node PP < 0.5/ BS<50%.

In the Caryophyllaceae tree, *Brachystemmacalycinum* and *Stellariaovatifolia* were nested in the tribe Alsineae with strong support (PP = 1.00, BS = 100) (Fig. 3A). Moreover, in the tree encompassing Alsineae tribe, *B.calycinum* and *S.ovatifolia* formed a monophylum (PP = 1.00, BS = 99) with strong support (PP = 1.00, BS = 100) (Fig. 3B), which is sister to the clade composed of *Schizotechium*, *Mesostemma*, *Lepyrodiclis*, *Shivparvatia*, *Odontostemma*, and *Pseudostellaria* in this tree (Fig. 3B). Our results suggested *Stellariaovatifolia* was closely related to *Brachystemma*, instead of either *Stellaria* s.str. or *Schizotechium*.

### ﻿Ancestral character

**Figure 4.**
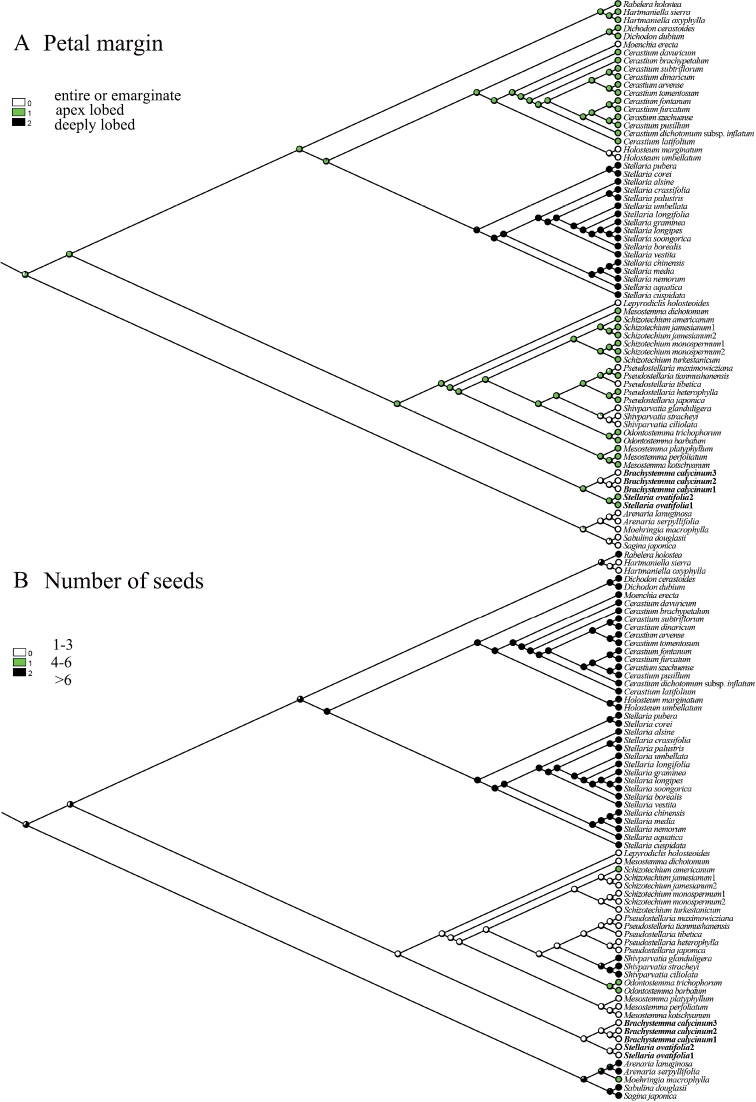
Evolutionary cladograms of the distribution of two character in Alsineae**A** petal margin **B** number of seeds.

The results of the ancestral character reconstruction indicated that petals with a lobed apex and numerous seeds may be the ancestral characters of the tribe Alsineae (Fig. 4). The presence of entire petals and 1–3 seeds became the diagnostic characters between *Brachystemma* and related genera. In addition, *B.calycinum* and *S.ovatifolia* shared the characters of 1–3 seeds and neither taxa has deeply bifid petals. It suggested a close relationship between *B.calycinum* and *S.ovatifolia*.

## ﻿Discussion

### ﻿Phylogenetic position and distinction of *Brachystemma*

As currently defined, *Brachystemma* is a monotypic genus in the tribe Alsineae, which is characterized by annual subscandent life form, lax thyrse with many flowers, petals shorter than 1/2 the length of the sepals with entire margins, two styles, four-valved capsules, and one mature seed (Fig. 1) (Lu and Gilbert 2001). Our phylogenetic results also revealed that *Brachystemma* formed a single branch with *S.ovatifolia* (Fig. 2 and Fig. 3) and demonstrated that *Brachystemma* is an independent genus (*S.ovatifolia* will be discussed in the following paragraphs), which is consistent with traditional morphological studies (Fenzl 1840; Bentham and Hooker 1862; Pax and Hoffmann 1934; Bittrich 1993; Lu and Gilbert 2001; Takhtajan 2009). Furthermore, the phylogenetic position of *Brachystemma* was nested in the tribe Alsineae and sister to the clade composed of *Schizotechium*, *Mesostemma*, *Lepyrodiclis*, *Shivparvatia*, *Odontostemma*, and *Pseudostellaria* (Fig. 3). Nevertheless, *Brachystemma* can be morphologically distinguished from the related genera of this clade. *Brachystemma* and *Lepyrodiclis* share characters such as annual life form, lax thyrse, and two styles, but *Brachystemma* differs from the latter by subscandent life form and four-valved capsules (Lu et al. 2001). It also can be distinguished from *Mesostemma*, *Pseudostellaria*, and *Schizotechium* by annual life form, petals with entire margins, lax thyrse, and two styles (Lu et al. 2001; Arabi et al. 2022). It can be clearly distinguished from *Shivparvatia* by annual habit, lax thyrse, and two styles (Lu et al. 2001; Keshav and Kumar 2015). Finally, it can be segregated from *Odontostemma* by lax thyrse, petals with entire margin and wingless seeds (Lu et al. 2001; Sadeghian et al. 2015).

### ﻿Character evolution

Our results indicated that petals with a lobed apex and numerous seeds may be the ancestral characters of the tribe Alsineae, which was consistent with previous studies (Greenberg and Donoghue 2011; Zhang et al. 2017). *Brachystemma* has entire petal margins, but it is sister to the clade composed of genera having lobed petals *Schizotechium*, *Mesostemma*, *Odontostemma*, and *Pseudostellaria* (except *Pseudostellariamaximowicziana* and *Pseudostellariatibetica*) (Fig. 4). Moreover, the tribe Alsineae is defined by a many-seeded (rarely few- or one-seeded) capsule or a rarely indehiscent nutlet (Harbaugh et al. 2010; Greenberg and Donoghue 2011; Arabi et al. 2022), but above genera having lobed petals share the character of fewer seeds (a capsule) (Fig. 4). The tribe Alsineae may have developed in an evolutionary direction toward fewer seeds. In addition, *B.calycinum* may be a species with diverse petals based on our field observations. *B.calycinum* may also include long (longer than sepals) and apically lobed petals (Fig. 1H), instead of only short (shorter than 1/2 the sepal length) and entire petals in the protologue (Fig. 1I). While additional observations in the field and specimens are required to confirm the petal condition, the petal condition in *Brachystemma* is coded here in accordance with the protologue.

### ﻿Classification of *Stellariaovatifolia*

Although the placement of *Stellariaovatifolia* among *Brachystemma*, *Schizotechium* and *Stellaria* has been uncertain for a long time, *S.ovatifolia* was considered more similar to *B.calycinum* in general appearance (Mizushima 1955; Wu and Ke 1996). It was clearly distinguished from the core *Stellaria* by subscandent life form (vs. non-scandent), lax thyrse (vs. cymes, rarely solitary), two styles (vs. three, rarely four or five), two-lobed (nearly to half of petal length) petals (vs. deeply-bifid petals), four-valved capsules (vs. six-valved capsules), and one mature seed (vs. many mature seeds) (Wu and Ke 1996; Lu and Gilbert 2001; Shilong and Rabeler 2001; Sharples and Tripp 2019). Despite being hypothesized to belong to *Schizotechium* (Pusalkar and Srivastava 2016), *S.ovatifolia* shows noticeable differences with *Schizotechium*, including a lax thyrse (vs. many-flowered compound cymes), two styles (vs. three styles), four-valved capsules (vs. six-valved capsules), and one mature seed (vs. one or two mature seeds) (Wu and Ke 1996; Shilong and Rabeler 2001; Pusalkar and Srivastava 2016). What is more, *S.ovatifolia* differs from *Brachystemma* by having two-lobed petals (nearly to half of petal length) and *Stellaria* type seeds, but they both share the following characters: subscandent life form, lax thyrse, two styles, four-valved capsules, and one mature seed (Fig. 1) (Wu and Ke 1996; Lu and Gilbert 2001; Shilong and Rabeler 2001). Hence, *S.ovatifolia* is highly similar to *Brachystemma*, instead of either *Stellaria* or *Schizotechium*. In terms of our molecular phylogeny, *Stellariaovatifolia* is nested with *Brachystemmacalycinum* in a clade with strong support (PP = 1.00, BS = 100) and not closely related to either *Stellaria* or *Schizotechium* in the nrDNA tree, cpDNA tree, and combined tree (Fig. 2 and Fig. 3). We believe that *S.ovatifolia* should be reclassified as a species of *Brachystemma* combining the evidence of similar general appearance and close phylogenetic relationship. As a result, the scientific name *Brachystemmaovatifolium* Mizushima is reinstated here. The main characters of *Brachystemma* now are: herbs annual or perennial; stems subscandent, branched; leaves opposite, petiolate; leaf ovate-lanceolate to lanceolate; stipules absent; inflorescence a thyrse or numerous in dichotomous, nearly subglobose cymes, terminal or axillary; flowers numerous, 5-merous, pedicellate; sepals free, subscarious, persisting in fruit; petals lanceolate or minute, much shorter than sepals, margin entire or bifid; stamens 5 or 10; styles 2; fruit a capsule, oblate, 4-valved, 1-seeded; seed reniform or globose.

### ﻿Taxonomic treatment

#### ﻿ Brachystemma

Taxon classificationPlantaeCaryophyllalesCaryophyllaceae

D.Don, Prodr. Fl. Nepal. 216. 1825. 

9589E181-278F-5397-928D-F48FD5A3CE92

##### Type:﻿

B.calycinum D.Don.

##### Two species.

*B.calycinum* D.Don, Prodr. Fl. Nepal. 216. 1825, and *B.ovatifolium* Mizushima, Acta Phytotax. Geobot. 16: 42. 1955.

## ﻿Conclusion

Based on our study, *Brachystemma* is clearly a separate genus nested in the tribe Alsineae and now includes two Asiatic species *B.calycinum* and *B.ovatifolium*. The native range of *B.calycinum* is Assam (India), Cambodia, South-West (Tibet, Xizang province) and South-Central China, East Himalaya, Laos, Myanmar, Nepal, Thailand, Vietnam (Wu and Ke 1996; Lu and Gilbert 2001; Shilong and Rabeler 2001). The native range of *B.ovatifolium* is Nepal and China (Tibet) (Wu and Ke 1996; Lu and Gilbert 2001; Shilong and Rabeler 2001).

## Supplementary Material

XML Treatment for Brachystemma

## Supplementary materials

Supplementary material 1 The vouchers detaled information Data type table (excel file) Explanation note The vouchers detaled information[DS/OL]. Science Data Bank, 2022[2023-02-02]. https://binary.pensoft.net/file/813066This dataset is made available under the Open Database License (http://opendatacommons.org/licenses/odbl/1.0/). The Open Database License (ODbL) is a license agreement intended to allow users to freely share, modify, and use this Dataset while maintaining this same freedom for others, provided that the original source and author(s) are credited.Wen-Qiao Wang, Zhong-Hui Ma
